# Thermodynamics of Binding Between Adeno-Associated Viruses and Heparin in Bulk and at Interfaces via Isothermal Titration Calorimetry

**DOI:** 10.3390/bioengineering13060631

**Published:** 2026-05-28

**Authors:** Elizabeth Adeogun, Jude C. Obijiaku, Ronny Horax, Kayla E. Daugherty, Joshua Sakon, Xianghong Qian, Barbara Knutson, Stephen E. Rankin, Karthik Nayani

**Affiliations:** 1 Department of Chemical Engineering, University of Arkansas, Fayetteville, AR 72701, USA; etadeogu@uark.edu (E.A.); jcobijia@uark.edu (J.C.O.); 2 Department of Biomedical, University of Arkansas, Fayetteville, AR 72701, USA; horax@uark.edu (R.H.); jsakon@uark.edu (J.S.); xqian@uark.edu (X.Q.); 3 Department of Chemical and Materials Engineering, University of Kentucky, Lexington, KY 40506, USA; kayla.daugherty@engr.uky.edu (K.E.D.); bknutson@engr.uky.edu (B.K.); stephen.rankin@engr.uky.edu (S.E.R.)

**Keywords:** isothermal titration calorimetry, AAV2, affinity-based separations

## Abstract

Adeno-associated viruses (AAVs) have emerged as promising vectors for gene therapy due to their non-pathogenic nature and ability to transduce various cell types efficiently. In recent years, there has been an increasing effort to optimize the production and purification of AAV to support clinical applications; however, challenges exist in affinity ligand design, synthesis, and characterization. Understanding the binding interactions of these viruses with functional molecules is pivotal for the development of affinity-based separation methods of AAVs. Classical methods to measure thermodynamic parameters such as Isothermal Titration Calorimetry (ITC) are challenging to employ in these scenarios, as the concentrations of the viral titers are significantly lower than those used in binding experiments with small biomolecules. Here, we present design principles that enable ITC-based determination of binding interactions between AAV2 and heparin. We observe increasing binding affinity with increasing molecular weight of heparin. We also elucidate the binding stoichiometry between AAV2 and heparins of varying molecular weights. Additionally, we report on the impact of buffer conditions and pH values on AAV2–heparin binding properties. Lastly, we also present the binding affinities and thermodynamic properties of interactions between the two species with heparin immobilized onto surfaces, namely, silica nanoparticles, as surface immobilization of the ligand is a common pathway for affinity-based separations. Overall, our results may provide key information for optimization of AAV-ligand binding protocols that are an essential step toward optimizing AAV capture and immobilization methods.

## 1. Introduction

The ongoing advancements in molecular biology have enabled the discovery of many viral vector therapeutics. Adeno-associated viruses (AAVs, the vector of choice in gene therapy) has continued to expand the possibilities for treating a wide range of diseases, including cancer, genetic, neurological, and infectious diseases [1,2,3,4,5]. The development of efficient unit operations for commercial-scale purification of virus particles remains a major challenge that often affects the purity and economic viability of virus-particle-based therapeutics [1,2,3]. For example, the recombinant adeno-associated virus (AAV) is a promising gene therapy candidate. Despite over 100 clinical trials involving AAV vectors, there are only a handful of FDA-approved products that use AAV serotypes. The ratio of empty-to-filled particles can be as high as 10:1 [6]. The presence of empty viral capsids reduces the effectiveness of the therapy by competing for cell-mediated processes, as well as being an impurity that could lead to stronger immune responses. Currently, full and empty viral capsids are separated using density-gradient ultracentrifugation, which is difficult to scale for batch operations during large-scale manufacturing. A key barrier for AAV-based gene therapy is the affordability of the drug product. For example, at $3.5 million per dose, Hemgenix is one of the most expensive drug treatments available. A large fraction of the cost involved with such therapies is in the manufacture and quality control of the AAV vectors. In the context of quality control, empty viral capsids can range from 20% to over 98% in vector preparations when produced by standard transfection production pipelines. Common semi-quantitative methods of surveying empty or partially empty particles employ electron microscopy and are time intensive [7,8]. More sophisticated methods employing charge detection mass spectrometry are extremely capable but not scalable for high-throughput screening of the cargo of AAV vectors [7,8,9,10]. Current techniques utilize affinity-chromatographic resins designed for AAV purification, which are costly and prone to fouling, increasing manufacturing expenses. Heparin affinity chromatography has long been established as an effective capture step for AAV purification, exploiting natural capsid interactions with heparan sulfate to enable single-step, scalable recovery with high yields and biologically active vectors [11]. However, its applicability is serotype-dependent and it may co-purify impurities, and engineering capsids used to confer binding can compromise vector potency, transduction efficiency, or production performance in practice today [11,12,13]. Hence, less expensive and more accessible ligands need to be developed to reduce the cost of gene therapy.

Various strategies have been developed to design and synthesize low-cost affinity ligands, including peptide-based and antibody-based affinity ligands [14,15,16]. Recent advances in the development of affinity ligands for virus capture utilize protein peptides that mimic the biorecognition activity of the AAV receptor (AAVR) and monoclonal antibody (A20). Peptide ligands have received a lot of attention due to their high selectivity and capacity with mild elution conditions, including the release of products upon exposure to mild pH, light, and salts. These ligands can be produced on a large scale rapidly and cost-effectively, helping to lower production expenses. The primary factor influencing the biorecognition activity of peptide-based ligands is the balance between enthalpic and entropic contributions to the free energy of the target–ligand interaction [17]. Current methods of estimating the binding free energy of AAV and peptide ligands, as well as molecular docking and simulation, struggle to accurately model complex interactions like solvation effects and entropy changes [18,19,20].

The development of experimental methods that can determine the thermodynamic parameters of interactions between AAVs and ligands is therefore of critical importance. Isothermal titration calorimetry (ITC) is an analytical technique that directly measures the thermodynamics of interactions in solutions [17,21,22]. It has become a benchmark for studying binding processes due to its high precision, sensitivity, and its capability for simultaneous determination of several thermodynamic parameters. ITC works by measuring the heat released or absorbed during a binding event, allowing for the determination of key thermodynamic parameters such as the binding affinity (K_d_), enthalpy (ΔH), entropy (ΔS), and the binding stoichiometry (n) of binding interactions. The combination of these interactions leads to the net exothermic or endothermic phenomena observed in the experiments. ITC has been used to measure the binding affinities of several biological, biochemical, and biophysical processes, including protein–ligand binding, protein–protein binding, protein–metal binding, and polyelectrolyte complexation [23,24,25,26,27,28]. Despite these successful applications, generating interpretable thermograms using ITC has certain criteria that need to be met, and this is often challenging when using binding studies where one of the species is present at a low concentration, such as viral titers. It is therefore no surprise that studies that report binding between virus and ligands via ITC are sparse [29].

Here, we present a pathway to overcome the challenges mentioned above and report on the thermodynamics and stoichiometry of interactions of AAVs with heparin using ITC. The role of chain length on influencing the thermodynamic parameters was studied by using different molecular weights of heparin. The role of pH was explored by dispersing vectors and ligands under different buffer conditions, as these present challenges to vector release during the downstream processing. Lastly, the interaction of heparin-functionalized silica nanoparticles with vectors was studied as an initial test for the feasibility of using this system for membrane-based purification systems.

## 2. Materials and Methods

### 2.1. Preparation of Heparin

Sodium salt of porcine intestinal heparin (150 units/mg) and porcine intestinal heparan sulfate were purchased from Sigma Aldrich (St. Louis, MO, USA). Heparin sodium salt (low molecular weight 65 units/mg) was obtained from MP Biomedical. Stock solutions were made in phosphate buffer (PBS, pH 7.4), and the concentration was derived based on reported molecular weights of each [MW (heparin) = 15,000 − 18,000, MW (heparin) = 3000]. Stock solutions of sodium acetate (pH 5.6) and glycine (pH 9.0) were prepared for the pH-dependent experiments. Solutions were mixed to obtain the required final concentrations of each component for individual experiments.

### 2.2. Preparation of AAV2

HEK 293 suspension cells were cultivated using a complete growth medium Gibco^®^ viral vector HEK medium containing 2% (*v*/*v*) GlutaMAX™ supplement (both purchased from Thermo Fisher Scientific (Waltham, MA, USA)). The medium was pre-warmed to 37 °C before inoculation. The initial cell seeding density was set at 0.3 × 10^6^ cells/mL. The culture was maintained in a CO_2_ shaking incubator at 37 °C and 82% relative humidity under continuous shaking at 250 rpm. When the cell density reached 5.0–6.0 × 10^6^ cells/mL, the culture was diluted with fresh medium to adjust the final cell density to 3.0 × 10^6^ cells/mL for AAV2 capsid production. AAV2 plasmids, including pAAV2 gene, pAAV-rep2/cap2, pHelper at 1.0:2.0:0.5 ratio, were transfected into the HEK 293 cells for AAV2 vector production [30]. The culture was incubated for 72 h before harvesting. During harvesting, a 10% (*v*/*v*) AAV-MAX lysis buffer was added to the cell medium and incubated for 4 h to lyse the cells and release AAV2 capsids into the medium. Tangential microfiltration using BioOptimal^TM^ MF-SL microfilter (purchased from Asahi Kasei Bioprocess America, Inc. (Glenview, IL, USA)) was performed to remove the cells and cell debris from the AAV2 lysate. Thereafter, affinity capture was performed with the clarified feed to further purify the AAV2 vectors.

### 2.3. Isothermal Titration Calorimetry (ITC)

ITC experiments were conducted at 25 °C on a Microcal VP-ITC Microcalorimeter (Equipconsult LLC, Boston, MA, USA). AAV2 and heparin solutions were prepared in pH 7.4 phosphate buffer (PBS). The AAV2 solution was placed in the sample cell, and the heparin titrant solution was loaded into the syringe injector. Titrant was added in 10 μL increments, with a 240 s delay between injections. Heat of reaction was measured after each addition of titrant. Calorimetric titration data were fit to give the reaction stoichiometry, reaction binding constant (K_d_), and the binding enthalpy (ΔH) using the Origin 5.0 nonlinear least-squares program supplied with the Microcal VP-ITC. The system also gave information on the change in entropy (ΔS). The reported parameters are the average of at least triplicate measurements. The effect of dilution of the heparin solution in the titration cell was controlled by performing a blank titration, which consisted of titration of heparin into buffer solution.

### 2.4. Synthesis of Heparin-Functionalized Silica Nanoparticles

The synthesis of SNPs was guided by the correlation developed by Bogush et al. [31] to produce particles with a narrow size distribution and desired diameter (in this case, 40 nm). In a round bottom flask, deionized ultrafiltered (UF) water and ammonium hydroxide were added to anhydrous ethanol to final concentrations of 35.9 M and 3.1 M (NH_3_). The silica precursor, TEOS, was added dropwise to a concentration of 0.4 M, and the solution was allowed to react for 6 h at room temperature with moderate agitation. Afterwards, the particles were centrifuged out of solution, washed with anhydrous ethanol to remove any unreacted precursor, and cured overnight at 200 °C.

SNPs were characterized via scanning electron microscopy (SEM, Figure S2) and dynamic light scattering (DLS, Figure S3). A sample was prepared for SEM by suspending the particles in anhydrous ethanol and dispersing them through sonication for 60 min. A drop of the particle solution was placed onto a plasma cleaned silicon wafer and sputter-coated with a 0.5–2 nm thick layer of platinum using a Leica EM ACE600 (Leica Microsystems Inc. (Deerfield, IL, USA)) carbon/sputter coater. The sample was then imaged with a FEI Helios Nanolab 660 (Thermo Fisher Scientific (Waltham, MA, USA)). To determine the hydrated diameter of the SNPs through DLS, SNPs were dispersed in UF water through sonication for 60 min. The solution was then pipetted into a quartz cuvette for measurements with an Anton Paar Litesizer 500 (Ashland, VA, USA).

### 2.5. Silica Nanoparticle (SNP) Functionalization and Quantification

SNPs were amine-functionalized with amines through pre-treatment, grafting, and post-treatment steps [32]. The particles underwent a hydroxylating pre-treatment, where they were submersed in a 50:50 (*v*:*v*) solution of methanol and water, whose pH was adjusted to 3 with nitric acid, at a concentration of 0.5 mg/mL. This was followed by sonication for 30 min prior to incubating the particle solution at 40 °C for 4 h with moderate agitation. Following hydroxylation, the SNPs were recovered with centrifugation, washed with anhydrous ethanol, and dried overnight at 80 °C. The dried SNPs were resuspended in anhydrous ethanol at 0.5 mg/mL and sonicated for 30 min. Under nitrogen, APTES was added dropwise to a final concentration of 5 μL/mL, and the reaction proceeded overnight at room temperature with moderate agitation. After the addition of amines, the amine-functionalized SNPs (amine-SNPs) were recovered with centrifugation and washed with anhydrous ethanol to remove any unreacted amines. Amine-SNPs were cured overnight at 80 °C.

### 2.6. Amine Quantification of Amine-SNPs

Amine densities on amine-SNPs were quantified with a colorimetric assay utilizing an anionic dye, AO II, and analysis with UV-Vis spectroscopy [33]. Amine-SNPs (5 mg) were suspended in 2 mL of a 0.5 mM AO II aqueous solution and titrated to pH 3 with HCl. The particle solution was sonicated for 30 min and then incubated at room temperature with moderate agitation for 90 min. Dye-loaded amine-SNPs were recovered through centrifugation and washed with 2 mL of pH 3 water (HCl). AO II was released by adding 2 mL of pH 12 water (NH_4_OH) to the particles, sonicating for 30 min, and incubating with moderate agitation for 15 min. The particles were recovered with centrifugation, and the resulting dye solution was analyzed with an Agilent BioTek Synergy H1 Plate Reader at 495 nm for comparison against a standard curve.

### 2.7. Heparin Functionalization of Amine-SNPs

Unfractionated heparin from porcine mucosa (MW 16.4 kDa) was immobilized on the surface of amine-SNPs through the formation of an amide bond [34]. Amine-SNPs were added to a 0.1 M MES buffer, synthesized using MES Monohydrate Ultrapure, at 4 mg/mL and sonicated for 30 min to disperse the particles. EDC and NHS were added to the particle solution at 1.6 mg/mL and 0.96 mg/mL, respectively, and dissolved with moderate agitation. Once the EDC and NHS were fully dissolved, heparin was added at 76 μg/mL. The solution was incubated overnight at room temperature with moderate agitation. Heparin-functionalized amine-SNPs (heparin-SNPs) were recovered with centrifugation, washed with UF water, and dried under vacuum at room temperature.

### 2.8. Quantification of Heparin Attachment to Heparin-SNPs

Heparin densities on heparin-SNPs were quantified with a colorimetric assay using a cationic dye, methylene blue (MB), and analyzed with UV-Vis spectroscopy [35]. Heparin-SNPs (2.5 mg) were suspended in 0.16 mM aqueous MB solution (pH 6) and sonicated for 30 min, followed by incubation at room temperature for 90 min with moderate agitation. Dye-loaded heparin-SNPs were recovered through centrifugation and washed with 2 mL of pH 6 water. Charge screening was employed to release the bound MB [36]. An amount of 2 mL of a 0.5 M aqueous NaCl solution was added to the particles, followed by sonication for 30 min and incubation for 45 min with moderate agitation. Heparin-SNPs were recovered through centrifugation, and the resulting dye solution was analyzed an Agilent BioTek Synergy H1 Plate Reader at 668 nm for comparison against a standard curve.

### 2.9. Capping of Amines of Heparin-SNPs with Terminal Carboxylate Groups

To increase colloidal stability, residual amines on heparin-SNPs were blocked with a terminal carboxylate group using an amine capping procedure [37]. Heparin-SNPs were suspended in 0.1 M sodium carbonate–bicarbonate buffer (pH 9) at 0.5 mg/mL and dispersed via sonicating for 60 min. Citraconic anhydride was added to the solution dropwise to maintain solubility at 10× the original amount of amines present, prior to heparin functionalization. The reaction took place at room temperature with moderate agitation for 60 min. The amine-capped heparin-SNPs (RCOO-Heparin SNPs) were recovered via centrifugation, washed with fresh buffer, UF water, and ethanol, and dried overnight at 80 °C.

### 2.10. Measurement of Zeta Potential

To compare the surface charges of particles and evaluate their colloidal stability, DLS was used to measure zeta potential (ζ) with an Anton Paar Litesizer 500. Using an Omega Cuvette, ζ measurements were conducted at pH 3, 6, and 10 for amine-functionalized SNPs, heparin-functionalized SNPs, and heparin-functionalized SNPs whose surface amines had been capped with terminal carboxylate groups. Solutions of the desired pH were created by adjusting DI water with HCl or NH_4_OH. Particles were suspended in the aqueous solutions at 0.5 mg/mL and dispersed with sonication for 60 min, prior to being pipetted into the Omega Cuvette.

## 3. Results and Discussion

### 3.1. The Concept of c-Value

**Figure 1:**
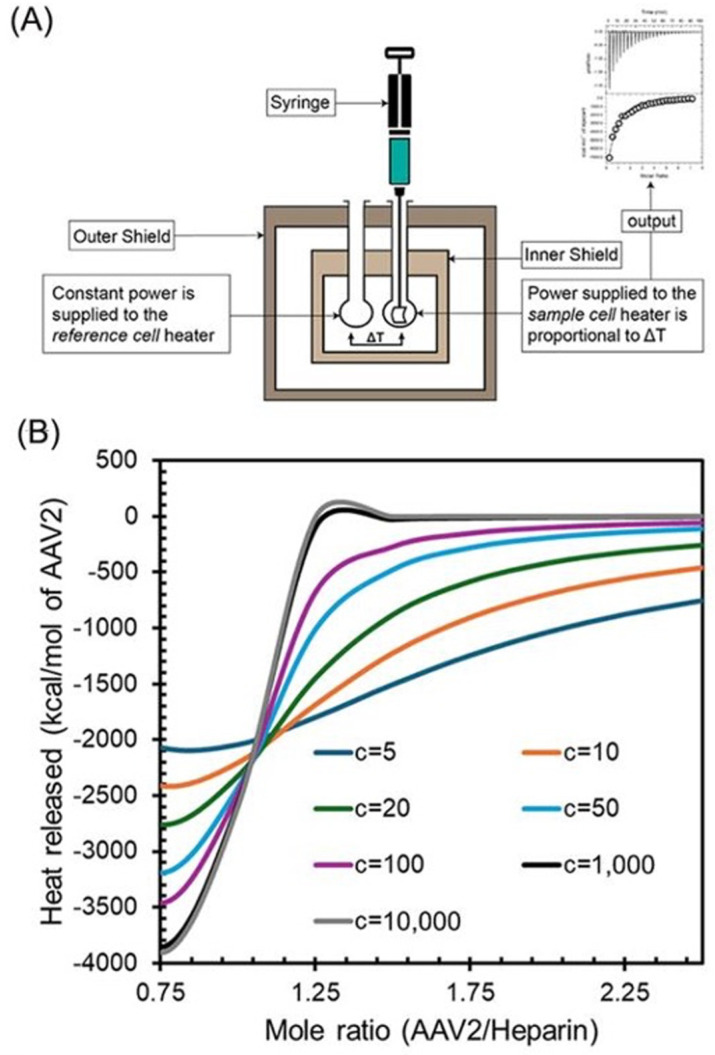
(**A**) Schematic of an ITC instrumentation showing sample and reference cells placed within an adiabatic jacket. (**B**) Simulated binding isotherm for a 1:1 macromolecule–ligand system showing importance of the “c” value.

A schematic of a typical ITC instrument is presented in Figure 1, wherein the sample cell and reference cell are placed within an adiabatic jacket. A key aspect of an ITC experiment is the choice of the right concentrations of the specimen in the sample chamber and the syringe. This is quantified by a quantity referred to as the “c” value, which is defined as:

c = nP_t_/K_d_ (1)

where n is the number of binding sites on the species in the sample cell, P_t_ is the concentration of the species in the sample cell (in the most common form of an ITC experiment, this species would be a protein), and K_d_ is the dissociation constant of reaction between the two species. Conventionally, for accurate determination of thermodynamic parameters and stoichiometry, the plot presented in Figure 1B should follow a clear sigmoidal behavior. Figure 1B plots simulated data of heat released as a function of ratio of the species for different c values. For this simulation, we assume values of K_d_, P_t_ and ∆H to be 1 nM, 30 nM and ∆H = −120 kcal/mol ligand, respectively. To generate the data for the simulated plot of Figure 1B, the proposed model of Wiseman et al. for a binding reaction with 1:1 stoichiometry, as described in Equation (2), was used.

(2) $$1/V_{0}\left(\frac{dQ}{dX_{tot}}\right)=∆H^{0}\left(\frac{1}{2}+\frac{1-\frac{1+r}{2}-\frac{X_{r}}{2}}{({{X_{r}}^{2}-2X_{r}\left(1-r\right)+{\left(1+r\right)}^{2})}^{\frac{1}{2}}}\right)$$

where is $X_{tot}\;$is the total ligand concentration (free plus bound) in the reaction volume $V_{0}$, Q is the heat absorbed or evolved, and the two unitless parameters r and $X_{r\;}$ both depend on the total ligand concentration and the total macromolecule concentration ($M_{tot}$) as follows:

(3) $$1/r=\;c\;=\;M_{tot}K$$

(4) $$X_{r}={X_{tot}/M}_{tot}$$

Values of c were set between c = 5 and c = 10,000, and Equation (3) was used to obtain the respective values of r for each c value. Also, the mole ratio $X_{r\;}\;$was plotted between values of 0.75 to 2.5, and Equation (4) was used to obtain values for total ligand concentrations at the various chosen mole ratios for each c value. An ITC sample cell volume of 1.45 µL was used as the reaction volume $V_{0}$.

We note from Figure 1B that c values between 20 to 100 result in desired sigmoidal shape of the curve, whereas both low and high values of c result in significant deviation from the desired shape. The challenge when working with viral samples with extremely low titer values (~10^−11^–10^−9^ M) is that the c values can be much below the desired range specified above (20–100). As Figure 1B depicts, low values of *c* (<10) result in a shape of the binding isotherm that does not permit accurate estimation of the thermodynamic parameters unless one of them is already known (stoichiometry, for instance) and is not varied during the fitting process [29]. To circumvent this challenge, we underscore a few key design criteria that would result in a c value that is as close as possible to the desired range: (1) The AAVs have to be placed in the sample cell as opposed to the syringe such that the maximal value on n is attributable to the computation of c value (for instance if the AAV has 10 binding sites, n = 10 if the AAV is in the sample cell as opposed to 0.1 when it is in the syringe). (2) The optimal range for K_d_ is in nM or lower regime, which implies ligands which have strong affinity for AAVs. A weakly binding ligand (K_d_~µM) would result in value of c that is well below 20 and therefore unreliable determination of thermodynamic parameters. We note that these design principles are generic and hold whenever ITC is used to determine the thermodynamics of interactions with the limitation being the low concentration of one of the species of interest. Data with low c-values are highly susceptible to experimental noise [38,39]. c-value optimization improves ITC robustness by enhancing signal-to-noise ratios and stabilizing the data fits. We have shown a decision tree that can help with the optimization of c values in cases with low titer values in Figure S1.

### 3.2. AAV2-Heparin Binding Interactions

Heparin, a highly sulfated glycosaminoglycan, serves as the model ligand in this study [40,41,42,43]. Heparin binds to AAV2 via electrostatic interactions of their dense negative charge to the positive amino acids on AAV2 capsids [41]. Bindings of heparin and AAVs have been previously characterized via techniques other than ITC and have been shown to have a strong binding affinity (K_d_~1–2 nM) [40,44]. Our attempts in this work to determine the binding constants, enthalpy changes, and stoichiometry of interactions via ITC revealed that several factors must be considered, such as the concentrations of AAV2, pH of buffers, the effect of surfaces onto which the ligand is immobilized, and most importantly optimization of ITC setup to get the desired “c” value range. Precise determination of the thermodynamic parameters governing the interaction of these virus vectors with their ligands is essential for optimizing ligand design, improving vector purification and processing, and enhancing the efficacy of vector-based gene therapies. Thus, once optimized, ITC can serve as a versatile tool for understanding and manipulating the interactions of AAVs with other ligands in the context of gene therapy applications.

**Figure 2:**
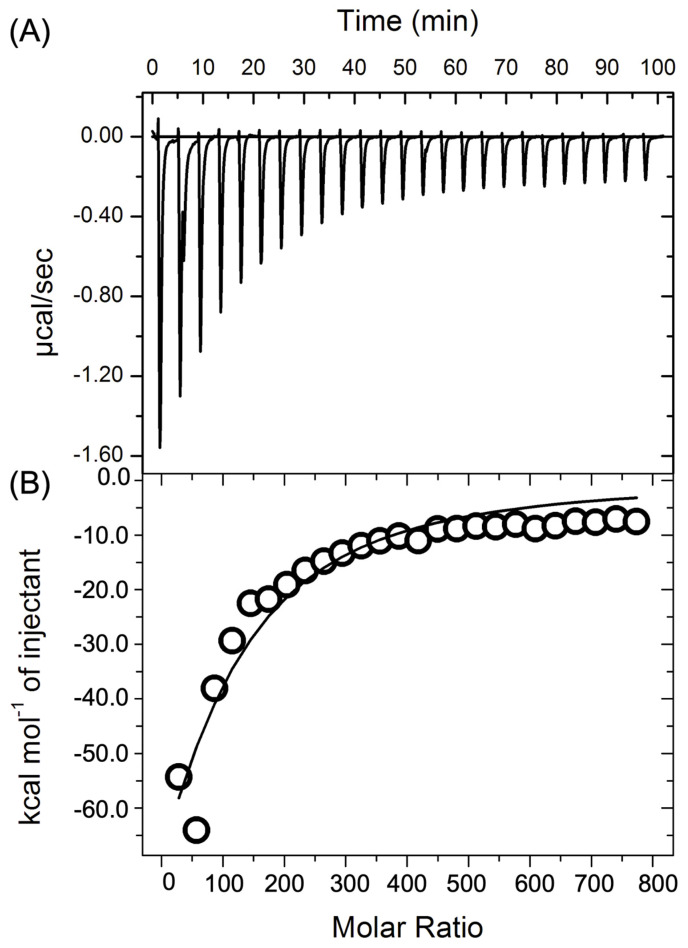
A typical ITC experiment. The experiment shown corresponds to the titration of 17.5 kDa heparin with AAV2. The experiment was performed in 1× PBS buffer, pH 7.4 at 30 °C. 17.5 kDa IN the heparin = 5 nM and [AAV2] ~ 0.1 nM. In an ITC experiment, the quantity measured and displayed on the *y*-axis is the time dependence of the electric power (μcal/s) necessary to maintain constant the temperature difference between the reaction and reference cells after each injection of reactant. The area under each peak is the heat (microcalories) associated with the process.

A typical binding isotherm for the interaction between AAV2 and 17.5 kDa heparin is shown in Figure 2. In this experiment, 1.4 mL of a 9 × 10^12^ vg/mL (vector genomes/mL) solution of the AAV2 was titrated every 240 s with 10 µL of a 5 nM 17.5 kDa solution. The peaks correspond to an exothermic reaction. The peak area diminished with each new injection of heparin, and the small peaks of similar areas near the end of the titration correspond to the heat of dilution for heparin. In a separate experiment, the heat of dilution for heparin was determined by injecting heparin into PBS buffer in the absence of AAV2. This constant was subtracted from the raw data (Figure 2A) to obtain the kcal of heat evolved from each injection (Figure 2B). The integrated peak areas were analyzed using an independent multiple binding sites model to determine the parameters ΔH (binding enthalpy in kcal/mol ligand), K_d_ (binding constant), and n (binding stoichiometry). The Gibbs free energy (ΔG) was calculated using the equation ΔG = −RTlnK, and the entropy (ΔS) was derived from the relationship ΔG = ΔH − TΔS. The AAV2 vector binds with strong affinity to heparin, as evidenced by a characteristic ITC thermogram presented in Figure 2.

Using a multiple site fitting model, we generated thermodynamic parameters for the interaction of 17.5 kDa and low molecular weight 3 kDa heparin with AAV2, as shown in Table 1. The enthalpy of interaction corresponding to the heat of injection was observed to increase with increasing molecular weight of heparin, while the dissociation constant reduces, thereby denoting a more robust interaction. The enthalpies of interaction measured during the titration of ligands into AAV2 were negative, indicating an exothermic interaction. Heparin (~17.5 kDa) carries greater number of negative charges (per mole) compared to the lower molecular-weight (LMW ~ 3 kDa) heparin, therefore, we see that the enthalpy of interaction is larger for the 17.5 kDa heparin. However, when normalized per negative charge, the binding enthalpies of the two heparins are similar. Measurements of K_d_ were also compared. Strong to moderate binding was observed for all AAV2-ligand interactions with K_d_ ranging from 2 × 10^−6^–0.9 × 10^−9^ M as presented in Table 1.

We note from Table 1 that the stoichiometry of binding between AAV2 and heparin is a function of the ligand size. When normalized by the number of charge units along the heparin chain for the respective molecular weights, we find that AAV2 is associated with 10.3 and 7.2 charged units of 3 kDa and 17.5 kDa heparin, respectively—this assumes a repeat unit weight of 0.6 kDa and 3 charges per unit. We also note that there is little change in the measured values of the Gibbs free energy for the different heparin molecular weights. This is consistent with the conventional understanding of enthalpy-entropy compensatory effects, wherein additional binding contribution are offset by a loss of confirmational entropy. Larger molecules would generally exhibit more favorable enthalpic contributions due to additional binding sites/contacts, but this is mostly offset by greater entropic losses due to reduced conformational freedom and multivalent binding constraints [45,46]. As observed in our AAV2-Heparin system (Table 1), these compensatory effects result in a minimal difference in the value of binding free energy changes across the varied molecular weights.

### 3.3. Role of pH

**Figure 3:**
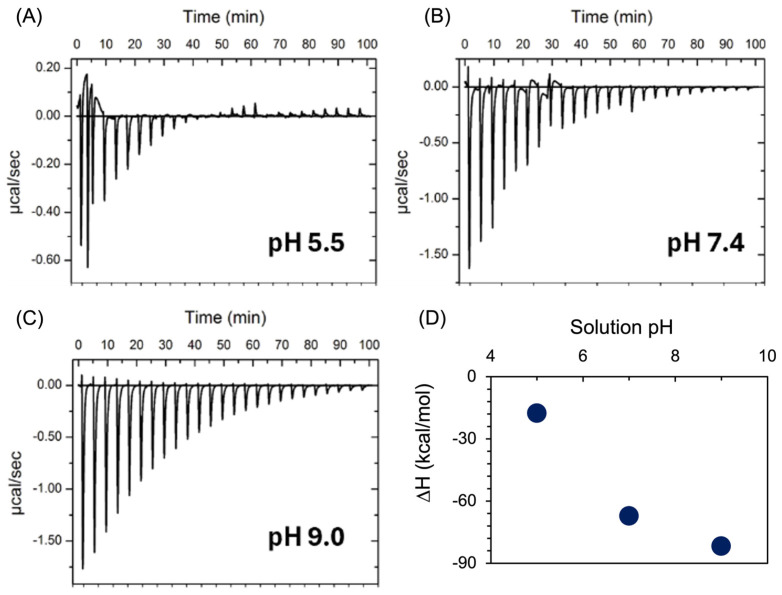
ITC isotherms of interaction between AAV2 and heparin in buffers of different pH (**A**) sodium acetate buffer pH 5.5, (**B**) phosphate buffer pH 7.4, and (**C**) glycine buffer pH 9.0. (**D**); pH dependence of ∆H for heparin-AAV2 binding.

The binding interaction of heparin with proteins is electrostatic; hence, the reaction of AAV2-heparin is expected to be pH-dependent. The heat effect associated with the binding of AAV2 to heparin has been measured in buffers of different pH (sodium acetate buffer pH 5.5, phosphate buffer pH 7.4, and glycine buffer pH 9.0) using ITC. Figure 3A–C represents the ITC profiles of AAV2-heparin (17.5 kDa) interactions and standard enthalpy changes as a function of pH, respectively. The changes in standard enthalpy as shown in Figure 3D suggest that the reaction of AAV2 and heparin becomes increasingly favorable with an increase in pH. The ΔH shows an abrupt change in going from −17.7 to −67.2 kcal/mol between pH 5.5 and 7.4. This indicates that ionization of the charged groups is significantly different at those to pHs.

### 3.4. Heparin Functionalized Silica Nanoparticles

**Figure 4:**
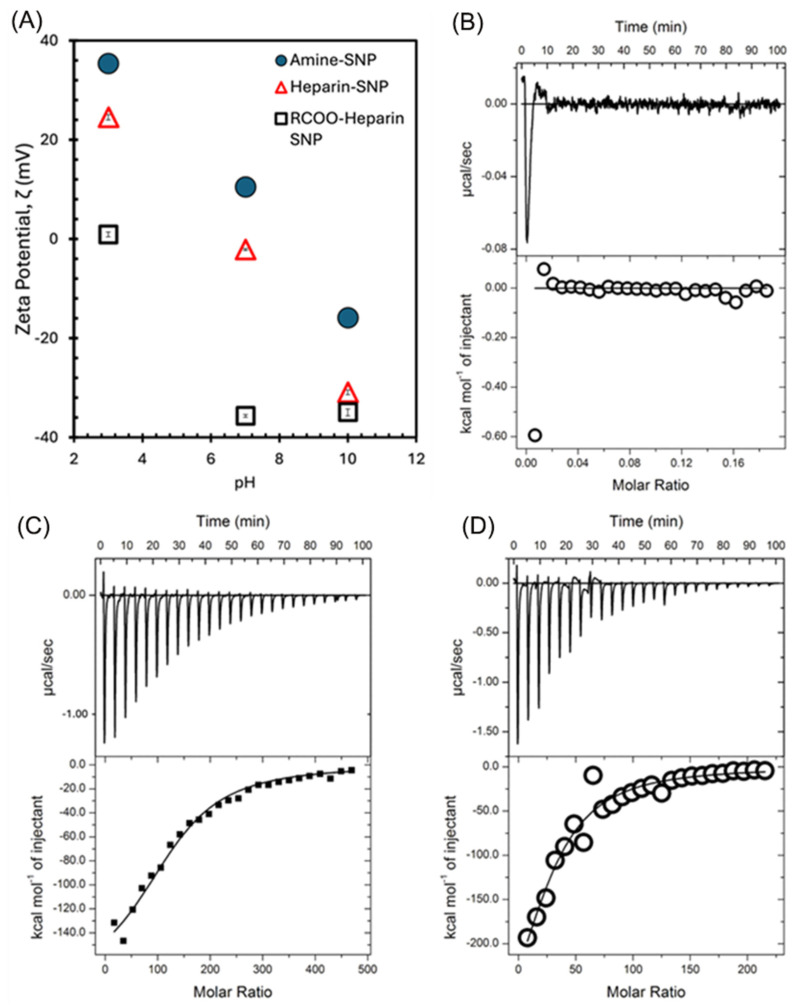
Binding interaction of heparin functionalized silica nanoparticles (SNPs). (**A**) Zeta potential distribution of amine-SNPs, heparin-SNPs and carboxylate-capped heparin SNPs (RCOO-Heparin SNP) dispersed in pH 3, 7 and 10 aqueous solutions. (**B**) ITC profile of RCOO-Heparin SNPs titrated into sample cell containing PBS buffer as a control experiment. (**C**) ITC profile of RCOO-Heparin SNPs (of ~17.5 kDa heparin) titrated into sample cell containing AAV2s. (**D**) ITC profile of 17.5 kDa heparin titrated into sample cell containing AAV2s.

We also studied the binding of heparin functionalized silica nanoparticles with AAV2s. Heparin was grafted to the amine-functionalized silica nanoparticles by creating amide bonds via the carboxylic acid groups of heparin silica EDC/NHS coupling. The combination of heparin with the silica nanoparticles (SNPs) should lead to an efficient model affinity ligand that can allow for developing methods for immobilizing the ligands to membranes and testing their performance. To compare the surface charges of particles and evaluate their colloidal stability, zeta potential measurements were conducted at pH 3, 6, and 10 for amine-functionalized SNPs, heparin-functionalized SNPs, and heparin-functionalized SNPs, whose surface amines had been capped with terminal carboxylate groups. (RCOO-Heparin SNPs). Amines on heparin-SNPs were blocked with a terminal carboxylate group using an amine capping procedure in the RCOO-Heparin SNPs, which increased their colloidal stability. Solutions of the desired pH were created by adjusting DI water with HCl or NH_4_OH. Particles were suspended in the aqueous solutions at 0.5 mg/mL and dispersed with sonication for 60 min. The solutions were pipetted into an omega cuvette for measurements using an Anton Paar Litesizer 500. As shown in Figure 4A, the addition of heparin decreased the zeta potential measurements at all pH but resulted in an unstable colloidal system at the pH of the ITC experiments due to low surface charge around pH 7. Capping the free amines on heparin-SNPs with terminal carboxylate groups provided colloidal stability by dramatically decreasing the zeta potential.

Heparin was quantified using the binding of cationic dye methylene blue (MB) at pH 6, followed by its release using charge screening in a 0.5 mM NaCl solution. MB has been used to quantify heparin in solution using shifts in the absorption wavelength caused by interactions with the heparin chain, but here, it was just used as a charge-based probe of sulfate groups. The measured density of MB bound is 0.29 ± 0.03 mmol/m^2^ on heparin-functionalized particles. Using this value, we estimated 3.9 mg heparin/g of SNPs.

The specific interaction of heparin at the outer surface of RCOO-Heparin SNPs with AAV2 was evaluated with binding experiments using ITC and presented in Figure 4B–D. When RCOO-Heparin SNPs were titrated into PBS buffer containing no AAV2s, we expectedly see no signal from the instrument, as shown in Figure 4B. The ITC isotherms in Figure 4C confirm binding of AAV2 to the RCOO-Heparin SNPs (dissociation constant K_d_ ~0.8 nM); the functionalized heparin yielded Mw ~17.5 kDa in these experiments. Interestingly, these measurements follow closely the results of the heparin solutions of the same Mw (unbound) as presented in Figure 4D, with the enthalpies of RCOO-Heparin SNPs being only slightly lower. These results indicate that heparin functionalized onto interfaces can still serve as an effective affinity-based separation ligand for AAV2s. In an actual affinity-based separation we expect transport restrictions and steric hinderance to play a role in the binding kinetics. However, as our ITC sample cell is constantly stirred, these factors are less influential.

## 4. Conclusions

In conclusion, we present design principles that allow for the characterization of binding affinities of viruses (at low concentrations) with ligands using ITC. A model ligand, heparin, was titrated against nanomolar concentrations of AAV2, and we were able to measure the binding constants, enthalpy changes, and stoichiometry of interactions. The dissociation constant reduces with increasing molecular weight, elucidating a more robust interaction of AAV2 with longer chain heparin. Also, the comparison of the binding of AAV2 to heparin 17.5 kDa and to the LMW heparin 3 kDa shows apparent differences in the enthalpic and entropic contributions. The enthalpy is larger for binding to the 17.5 kDa heparin than for binding to the 3 kDa heparin; however, when normalized for the repeat units, the enthalpy to interactions are similar indicate a same dominant mode of binding, namely, electrostatic. By systematically adjusting the pH of the buffer solution while keeping other experimental conditions constant, we were able to uncover pH-induced shifts in the enthalpy associated with heparin-AAV2 binding. Here, the AAV2 and heparin interaction becomes increasingly favorable with increasing pH. Lastly, the binding affinities and thermodynamic properties of interactions between heparin-functionalized silica nanoparticles with AAV2 were investigated. We successfully used ITC to determine binding affinities between AAV2 and these modified surfaces and found that the heparin-functionalized silica nanoparticles have similar binding affinities as pure heparin of the same concentration, indicating that heparin immobilized on surface can still be effective for affinity-based separations of AAVs. The methodology identified here is general and therefore is applicable to other serotypes. However, some limitations do exist: (1) We did not perform advanced modeling of the ITC data to differentiate between cooperative binding and multiple independent modeling, among others. (2) For the experiments pertaining to immobilized heparin ligands on the surface of silica particles, diffusion limitations, steric constraints, etc., can influence the thermodynamic variables as compared to our system, wherein the species were constantly stirred within the ITC chamber. (3) We did not account for the polydispersity in the heparin molecular weights in the determination of stoichiometry.

## Figures and Tables

**Table 1:** Thermodynamic parameters for association of heparin and AAV2.

	Heparin (~17.5 kDa)	LMW Heparin [3 kDa]
ΔH° (kcal mol^−1^)	−67.2 ± 7.1	−12.9 ± 3.3
K_d_ (nM)	0.9	2
ΔG° (kcal mol^−1^)	−13.2 ± 3.4	−13.5 ± 1.8
ΔS° (kcal mol^−1^K^−1^)	−0.18	2 × 10^−3^
n (per heparin charge unit)	7.2	10.3

## Data Availability

The original contributions presented in the study are included in the article, further inquiries can be directed to the corresponding author.

## Supplementary Materials

The following supporting information can be downloaded at: https://www.mdpi.com/article/10.3390/bioengineering13060631/s1. Figure S1: Decision chart to help with c value optimization; Figure S2. (a) SEM of nonporous silica nanoparticles. (b) Particle size distribution determined using ImageJ software (version 1.54p) using a sample size of 1200. The SNPs are 40.6 + 6.3 nm in diameter: Figure S3. DLS analysis of hydrated nonporous silica nanoparticles. The SNPs are 50.3 + 11 nm in diameter.
