# Supplementary material for: Thermodynamics of Binding Between Adeno-Associated Viruses and Heparin in Bulk and at Interfaces via Isothermal Titration Calorimetry
Source: Bioengineering (Basel). 2026 May 28;13(6):631. doi: 10.3390/bioengineering13060631 (PMC13295711; doi:10.3390/bioengineering13060631)
Supplement: Supplementary file 1 [file bioengineering-13-00631-s001.zip › bioengineering-4275754-supplementary.pdf]

Supplementary Materials:

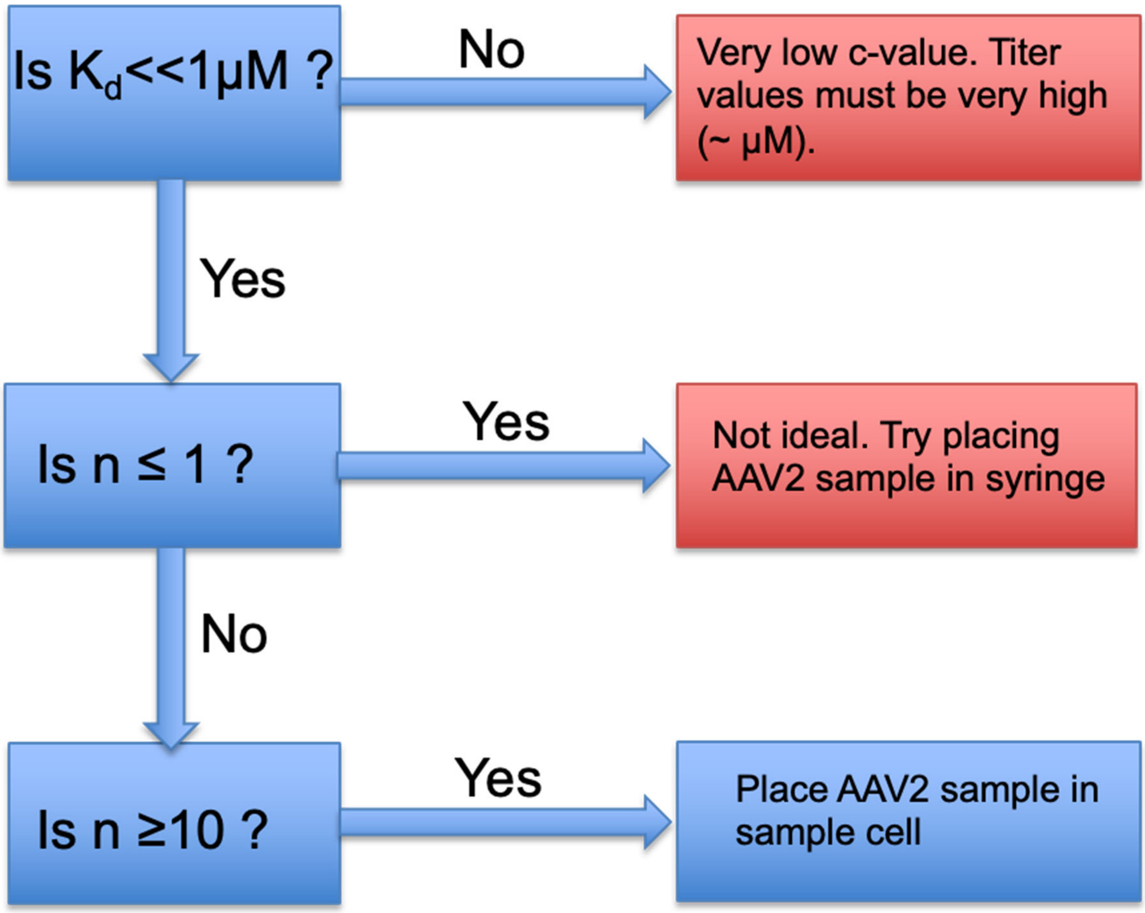

Figure S1: Decision chart to help with c value optimization

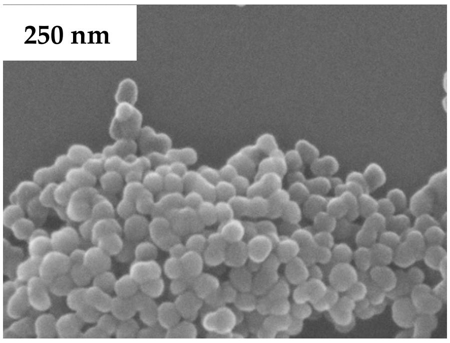

(**a**)

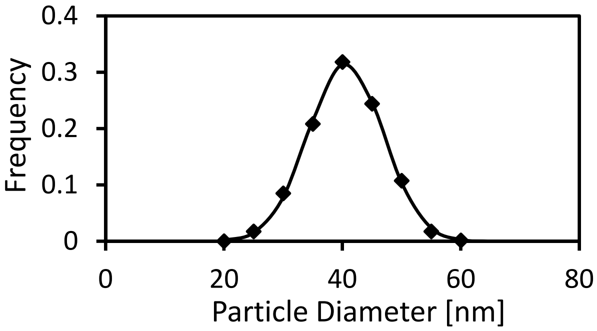

(**b**)

Figure S2. a) SEM of nonporous silica nanoparticles. b) Particle size distribution determined using ImageJ software using a sample size of 1200. The SNPs are 40.6 ± 6.3 nm in diameter.

Figure S2 shows the scanning electron microscopy images of nanoporous silica particles as synthesized by the methods we described above. Quantification if the particle size distribution is presented in Figure S2b.

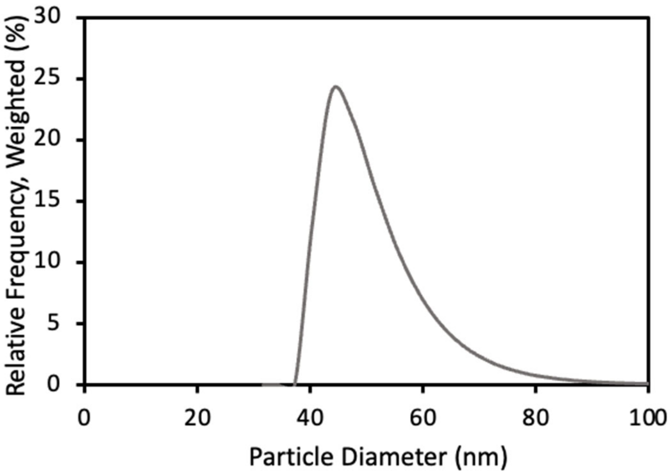

Figure S3. DLS analysis of hydrated nonporous silica nanoparticles. The SNPs are 50.3 $\pm$ 11 nm in diameter.
